# Deepfake forensics analysis: An explainable hierarchical ensemble of weakly supervised models

**DOI:** 10.1016/j.fsisyn.2022.100217

**Published:** 2022-01-27

**Authors:** Samuel Henrique Silva, Mazal Bethany, Alexis Megan Votto, Ian Henry Scarff, Nicole Beebe, Peyman Najafirad

**Affiliations:** a Secure AI & Autonomy Laboratory, Department of Electrical and Computer Engineering, University of Texas at San Antonio, San Antonio, TX, USA; b Secure AI & Autonomy Laboratory, Department of Information Systems and Cyber Security, University of Texas at San Antonio, San Antonio, TX, USA; c Department of Information Systems and Cyber Security, University of Texas at San Antonio, San Antonio, TX, USA; d Secure AI & Autonomy Laboratory, Department of Electrical and Computer Engineering, Department of Information Systems and Cyber Security, University of Texas at San Antonio, San Antonio, TX, USA

**Keywords:** Deepfake, Forensics, Deep learning, Facial recognition

## Abstract

Deepfakes have become exponentially more common and sophisticated in recent years, so much so that forensic specialists, policy makers, and the public alike are anxious about their role in spreading disinformation. Recently, the detection and creation of such forgery became a popular research topic, leading to significant growth in publications related to the creation of deepfakes, detection methods, and datasets containing the latest deepfake creation methods. The most successful approaches in identifying and preventing deepfakes are deep learning methods that rely on convolutional neural networks as the backbone for a binary classification task. A convolutional neural network extracts the underlying patterns from the input frames. It feeds these to a binary classification fully connected network, which classifies these patterns as trustworthy or untrustworthy. We claim that this method is not ideal in a scenario in which the generation algorithms constantly evolve since the detection algorithm is not robust enough to detect comparably minor artifacts introduced by the generation algorithms. This work proposes a hierarchical explainable forensics algorithm that incorporates humans in the detection loop. We curate the data through a deep learning detection algorithm and share an explainable decision to humans alongside a set of forensic analyses on the decision region. On the detection side, we propose an attention-based explainable deepfake detection algorithm. We address this generalization issue by implementing an ensemble of standard and attention-based data-augmented detection networks. We use the attention blocks to evaluate the face regions where the model focuses its decision. We simultaneously drop and enlarge the region to push the model to base its decision on more regions of the face, while maintaining a specific focal point for its decision. In this case, we use an ensemble of models to improve the generalization. We also evaluate the models’ decision using Grad-CAM explanation to focus on the attention maps. The region uncovered by the explanation layer is cropped and undergoes a series of frequency and statistical analyses that help humans decide if the frame is real or fake. We evaluate our model in one of the most challenging datasets, the DFDC, and achieve an accuracy of 92.4%. We successfully maintain this accuracy in datasets not used in the training process.

## 1 Introduction

Image forgery has taken the internet community and social media applications by storm in recent years. Specifically, utilizing deep learning (DL) approaches to learn specific image features and transpose them onto another image or video has become a sensation on applications like Snapchat, Instagram, Facebook, Reddit, and many others. This concept is otherwise known as “deepfake”, where “deep” is representative of the use of DL neural networks (NN), and “fake” represents its definition of being disingenuous to the original input.

Realistic video dubbing of foreign films [1], digitally reanimating historical figures [2], and using virtual fitting rooms to try on clothes [3] are just a few examples of how image forgery has become integrated with modern society. This imagery forgery is otherwise known as “deepfake”, in which “deep” refers to the use of deep learning (DL) neural networks (NN). Image forgery has also social media by storm in recent years. Apps like Snapchat, Instagram, Facebook, and Reddit employ DL approaches to learn specific image features and transpose them onto another image or video. These deepfake advancements have highlighted the potential implications of digital facial manipulation. Although entertaining and convenient, there exists a blatant and dangerous threat of malicious actors using deepfake attacks to jeopardize the safety of others.

In 2017, the user ‘deepfakes’ from Reddit used deep learning algorithms to exploit celebrity women by swapping their faces into pornographic videos and publicly posting them online.^1^ Defaming those celebrities and sparking controversy [4]. This incident created a media frenzy, inspiring new deepfakes. For example, BuzzFeed (an online media platform) released a deepfake video of former President Barack Obama providing a speech on the subject. Despite how closely the video resembled President Obama, it was a deepfake manipulation [5]. As sophisticated as this deepfake was, it was made possible by a public software called FakeApp [6]. The release of the app brought up several concerns about impersonation, the spread of misinformation on social media, and identity theft. These deepfakes have gained immense interest in the academic community. In its brief existence, deepfakes have raised concerns on the safety and security of innocent people. In March 2021, Google Scholar reported that the number of papers published on this subject rose from 11 in 2017 to 3,790 in 2021. Scholars are focusing immense energy on understanding and mitigating the threats of deepfake technologies.

Before the popularity of DL methods, image manipulation in fake image creation could produce realistic results. Yet this method relied on highly skilled image editing, and even so, a lot of time. DL apps such as FakeApp [6], FaceApp,^2^ and ReFace^3^ made the creation of deepfakes as easy and fast as a couple touches on a screen. These apps make use of mobile-optimized versions of DL models, such as Generative Adversarial Networks (GANs) [7], and Variational Auto-Encoders [8]. Among popular methods, FSGAN [9] is a GAN-based method used to generate face swapping in images and videos. FSGAN is independent of the subject and does not need subject-specific re-training, making it a perfect tool to be implemented in mobile devices. Moreover, in Ref. [10], the authors present a method based on VAEs, which can disentangle and identity feature representations in high-dimensional spaces. This makes it possible to modify someone's hair, pose, background and lighting, just based on reference sources. Even few-shot learning applies in the generation of deepfake samples to minimize the data required and make it even easier to generate deepfakes [11,12].

Although many deepfake generation models produce indistinguishable reproductions, these fakes can still be detected either by specialized forensic techniques [13] or deep learning methods [14]. Almost every deepfake generator leaves traces of its convolution operations in the image. While these traces can be detected by a careful forensic analysis [13], the current volume of data uploaded to social media necessitates fast and automatized tools. The sheer amount of data involved in analyzing deepfakes makes it impossible to conduct forensics analysis if a system does not establish automatic triage. If the user provides enough training data, supervised deep learning methods can easily detect the convolutional traces left in deepfake images. In Ref. [15], the authors proposed a DL method based on a relatively small network called MesoNet to detect whether a video is fake. In their work, the authors perform a frame-by-frame analysis to detect deepfakes.

Moreover, in Ref. [16], the frames’ optical flow is used for classification. A CNN was trained with the gradient of adjacent video frames to catch abrupt changes within the video. These abrupt changes showcase how deepfake videos generate in a frame-by-frame schema. As pointed out in Ref. [17], a wide variety of DL-based deepfake detection methods are subject to the representation of the dataset used for training. If a detector did not train with data created by a specific deepfake algorithm, it would likely have lousy accuracy when facing such a method after deployment.

The poor performance of DL methods when faced with unseen data, and their lack of explainability, reduces the confidence of specialists and security authorities. In this work, we propose an attention-based explainable deepfake detection algorithm. We address the generalization issue by implementing an ensemble of standard and attention-based data-augmented detection networks. We use the attention blocks to evaluate the face regions where the model focuses its decision. We simultaneously drop and enlarge the region to push the model to base its decision on more regions of the face, while maintaining a focus point for its decision. In this case, we use an ensemble of models to improve the generalization. We also evaluate the model's decision using a Grad-CAM explanation with a focus on features and attention maps [18]. The combined analysis of feature and attention heat maps allows forensic specialists to identify the location most relevant to the model's decision. Below is a summary of our contributions:

● We propose an ensemble of models for deepfake image and video detection to aid forensics research with attention-based data augmentation, thus improving our model's accuracy and generalization.
● We applied explanation techniques to demystify the machine's decision process by deriving a heat map of the regions in the video that most likely contributed to the model's decision, increasing the transparency in our model's prediction.
● We trained and evaluated our model with the Deepfake Detection Challenge, composed of more than 100 000 videos generated in the most adverse conditions.

The remainder of this paper is organized as follows. Section 2 offers a brief review of Deepfake literature pertinent to our problem. We highlight deepfake creation and detection methods, in order to understand how our approach differs from previously proposed approaches. Section 3 describes the architecture of our proposed model. Section 4 presents the experiments and results obtained by our attention-based explainable deepfake detection method. Finally, Section 5 concludes this paper with remarks on this study's results and prospective research opportunities.

## 2 Background

### 2.1 Overview

Digital face manipulation methods have evolved quickly, and continue to create new obstacles for forensic investigations. This section provides a thorough explanation of the four primary deepfake categories: *synthesizing, editing, reenactment, and replacement* [19,20]. Then, we will provide insight on the creation of deepfakes and conclude this section with an overview of two deepfake counter-measures that exist.

#### 2.1.1 Categories of deepfake

The first of these categories are *synthesizing* deepfakes, referring to deepfakes that create a non-real face by utilizing robust Generative Adversarial Networks (GANs) [14]. This deepfake technique has popularized rapidly as publicly accessible databases (100K-Faces, iFakeFaceDB) have provided researchers and hobbyists with the necessary resources to generate high-quality and realistic never-before-seen images. This category benefits the video game industry and 3D-modeling companies as well as malicious opportunists interested in spreading misinformation [14].

While synthesizing deepfakes focus on generating new images from learned facial characteristics, *editing* deepfakes secure the identity of the target image while facial and feature characteristics are changed. These deepfakes can learn facial attributes (hair, skin, eyes, and others) and how they are composed, allowing the user to manipulate these images to achieve specific goals. These include cosmetic adjustments, changing one's perceived age (appearing older or younger), and virtually trying on articles of clothing. While the beauty industry widely uses this technology, malicious online behavior also utilizes these capabilities. For instance, sexual predators use the same editing process to change their appearance in order to lure underage victims. Even non-malicious software that allows people to virtually try on clothes offers opportunists a platform to strip the users nude, allowing them to blackmail the users or spread fake images across the internet [2].

**Fig. 1:**
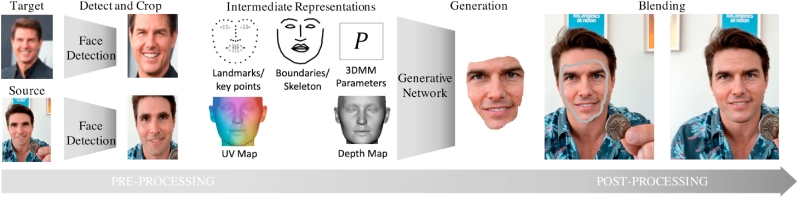
The process for reenactment/swap deepfake creation on human faces: (1) source and target faces are cropped and key features are extracted; (2) a generative network generates the target face driven by the source, and; (3) a post-processing blending is used to eliminate artifacts.

The third deepfake category is *reenactment*, which, as summarized in Fig. 1, uses a source face to inspire the changes of the target's face. Such changes include the target's expressions, gaze, mouth, pose, or body. Reenactment deepfakes allow attackers to manipulate the digital content through the impersonation of the victim's identity [21,22]. With available video and image resources, the potential attacker can leverage GANs to digitally regenerate an individual with the ability to control their speech, expressions, and mannerisms. This deepfake category creates dangerous opportunities for attackers to tamper with potential legal evidence or defame an individual, potentially destroying their reputation [6].

The most common deepfakes, however, are *replacement* deepfakes, which are sub-divided into two types, transfer and swap. Transfer replacement deepfakes digitally replace a target's content with another source. Face transfers are widely used by the fashion industry to assist customers with visualizing how they will look in an outfit before purchasing it [2,3]. The second replacement technique, swap, refers to the ability to digitally insert content from a source to a specified target while allowing the specified target to drive the change [23]. This technique is very popular for creating satirical content, such as memes. The swapping technique has also been used to replace one celebrity's face for another in different forms of media [1,24]. Replacement deepfakes have daunting implications, as they provide attackers with a range of harmful applications. This includes “revenge porn” (i.e. swapping the face of a victim over the face of a porn actor), and distributing fake news to spread misinformation (i.e. swapping the face of an important political figure to influence change).

Given the particular ubiquity and danger of reenactment and replacement deepfakes, this paper will provide an in-depth overview of those two techniques, as well as the detection methods available to combat this media. Unfortunately, identifying and locating fake videos has become increasingly difficult as attacks have become increasingly sophisticated. We will outline our novel detection tool in Section 5.4.

### 2.2 Deepfake creation

The most popular architectures used to create deepfakes are called *Encoder-Decoder Networks* and *Generative Adversarial Networks* (GANs). The first architecture, an encoder-decoder (ED) neural network, consists of a pair of concatenated neural networks with a bottleneck in the center. An image is processed through the encoder, where the network breaks down the image by pixel and learns its unique features. The encoder then processes the data through the bottleneck as an embedding (summary of concepts the network learned), and the decoder reconstructs the image based on the data in the embedding [25]. Of the two architectures, ED is the most popular for replacement deepfakes [26].

The second architecture is called a Generated Adversarial Network (GAN). This structure consists of a pair of neural networks, a generator, and a discriminator, which work against each other. The generator learns to create realistic samples of the data to fool the discriminator. The discriminator also learns to recognize if the input is fake or real. The discriminator releases an output called the generator loss, which creates a more comprehensive feature loss for the generator. As this back and forth process ensues, the zero-sum game leads the generator to create samples that are nearly indistinguishable from the actual sample distribution, that which the discriminator is trained on [26,27].

While GANs have advanced significantly, their ability to generate realistic deepfakes is still somewhat limited. Research conducted by Ref. [28] demonstrates that many deepfake datasets suffer from low visual quality. Considering deepfake algorithms are based on generative networks, they require copious amounts of data from both the target and the source (ex: thousands of pictures in various angles and lighting). Generally, it is much easier to obtain a large amount of data from the source. Because of this, recent research efforts have explored minimizing the required amount of target training data. Moreover, acquiring trained data is a highly laborious process which adds to the difficulty of creating convincing deepfake algorithms [29].

### 2.3 Deepfake countermeasures

As deepfakes threaten the public's safety and even national security, developing deepfake countermeasures is a matter of urgency among cyber security scientists. There are two available countermeasures: *prevention* and *detection*. Preventative countermeasures disrupt attacks through data provenance, data flow disruption, or direct model attacks [30]. Detection countermeasures detect and delete threats as they spawn on social media. Typically, this method leverages artifacts within a deepfake image to binarily classify whether an image is fake or real [31].

#### 2.3.1 Prevention

Preventing deepfakes has become an increasingly difficult challenge given their increasing sophistication. Yet, as deepfakes have advanced, so have preventative methods. One preventative measure uses the adversarial attacks to disrupt the specific network creating the images [28]. While this prevention method is a strong countermeasure, it does not generalize to unseen networks. Moreover, in Ref. [32], the authors propose using adversarial noise to prevent attackers from conducting face identification and gathering data for their malicious behaviors. This method reduces the amount of data available online for the attackers to generate deepfake images or videos. Although these approaches are very effective in disrupting deepfakes, an attacker can circumvent these methods with adversarial training and data augmentation. [33], on the other hand, proposes data provenance, which uses distributed ledgers and blockchain networks. A similar approach exists in Ref. [4], however, this approach focuses on smart contracts in an ethereum chain. Deepfakes can be spotted with relative ease by non-experts, depending on the generation method used. However, as deepfake software advances, deepfakes become increasingly more difficult to detect by the untrained eye. Therefore, it is critical that countermeasures are developed in order to gain an edge in this virtual arms race.

#### 2.3.2 Detection

Detection countermeasures provide an efficient solution for identifying impossibly realistic deepfakes. This method targets artifacts (specific characteristics within an image) to help determine whether or not it is fake. There are seven artifacts to help identify deepfakes from genuine content: *blending*, *environment*, *forensics*, *behavior*, *physiology*, *synchronization*, and *coherence*.

*Blending* is a technique used in deepfake methods to digitally blend the image into the frame. Researchers have proposed various mechanisms to assist in detecting whether an image is blended or not. For instance, the introduction of edge detectors, quality measures, and frequency analysis in recent literature assists in identifying blending artifacts [21,23,27,34,35].

Detection methods also analyze the *environment* for proof of a deepfake. For example, an allegedly fake face may present different pixel distribution compared to the background or other parts of the frame. These models search for patterns left by face warping algorithms [24,36], patterns introduced by light adjustments [37], and consistence [22], that may indicate the presence of fake content.

Thirdly, detection methods gather *forensics*. Forensic analysis includes identifying minor details introduced by attackers in the image. For example, GAN generated images carry a unique fingerprint [38,39]. In both publications it is indicated that it is possible to discriminate which algorithm has generated such forgery based solely in the pixels of the image. Compression does not mask these fingerprints. Even a camera's intrinsic parameters can be used to detect fake content [40].

The fourth artifact of deepfake content is *behavior*. If a large dataset is available from a specific target, it is possible to learn one's mannerisms and other involuntary behaviors. Even though it would not be directly applied to an average individual, celebrities and influential people could be protected from deepfake attacks, as shown in Ref. [41].

Countermeasures also study the *physiology* of an individual in an image or video. A series of physiological attributes can be monitored to discriminate deepfakes. For example a person's heart rate can be inferred from videos and used to discriminate between fake and real images [42], even irregular blinks can be exploited to identify forgeries [43]. In Ref. [44] the authors use the pulse extracted from the video as a feature for deepfake detection.

The sixth artifact of a deepfake attack can be found in the *synchronization* of the image content [45,46]. noticed that one can detect video dubbing attacks by matching speech and the movements of muscles associated with the mouth. Moreover, the method proposed by Ref. [47] match the phonemes and mouth shapes, to discern fake from pristine media.

The final artifact is content *coherence*. It is challenging to generate an image that maintains cohesion among its pixels, it is even harder to maintain the cohesion among adjacent frames. A series of authors exploits the temporal inconsistency that eventually arises on deepfake videos. For example [48], uses a Recurrent Neural Network (RNN) to detect artifacts such as flickers and jitter, and [26] uses recurrent networks on the face region only. In Ref. [49], the authors train a classifier on sequential pair frames. Furthermore, the authors in Ref. [16] improve the network's attention by evaluating the optical flow. The same researchers [50], based on the temporal coherence hypothesis, trained a network for next frame prediction. The deepfake detection is based on the comparison between the predicted frame and the actual frame. When the difference is above a specified threshold, the frame is marked as fake.

### 2.4 Undirected approaches

In deepfake detection, undirected approaches do not focus on specific aspects of the video. In order to train these particular architectures, the user must provide frames or videos as a whole rather than as specific frames. Undirected approaches use one of two methodologies: *classification* and *anomaly detection*.

*Classification*: the research presented in Refs. [19,51,52], clearly demonstrates that deep neural networks outperforms traditional forensics in deepfake detection. The evolution of the utilization of neural networks within forensics analysis has led to various authors demonstrating how standard CNN architectures can detect deepfake videos [15,[53], [54], [55]].

In [56], the authors approach the deepfake detection problem using Siamese networks. The authors train the networks by contrasting real and fake images. A natural concern in deepfake detection with CNNs is the potential for overfitting and lack of generalization to other attacks. In Ref. [57], the authors constructed a Hierarchical Memory Network (HMN) to mitigate the problem of generalization. The proposed method preserves the characteristics of previously processed faces in the memory. This network encodes the facial region and then processes it using a bidirectional gated recurrent unit. The encoded face is compared to recently evaluated faces and the model predicts the result based on the comparison between the recently seen faces and the currently encoded face. To improve model generalizability, the authors of [58] use an ensemble of 7 deepfake detection CNNs and plug in their outputs into a meta-classifier. With their approach, the number of false positives for deepfake detection reduced significantly compared to the use of individual models. Lastly, the authors of [59] evaluated a series of spatio-temporal approaches combined with different features extractors for deepfake detection. They found that RNNs and ID3 are outperformed by 3D CNNs.

From another perspective, *anomaly detection* algorithms are trained solely on real images, and are expected to detect the out of distribution samples at inference time. By doing so, this approach is not biased towards specific attacks. As a consequence, these methods are less biased towards specific attacks. The authors of [60] evaluate the neural activation of a face recognition network. Based on the results of obtained, the authors could clearly see that unaltered pixels had stronger signals than fake ones.

Similarly, in Ref. [61], the authors train a one-class Variational Autoencoder (VAE) to reconstruct authentic images and compute an anomaly score. To calculate this score, the VAE takes the Mean Squared Error (MSE) from the input and the reconstructed image. The authors of [10] evaluate the distance in the latent representation space. While [60,61] uses the model's shift due to distribution changes [10], evaluates the difference between extracted representations.

### 2.5 Datasets for undirected approaches

In order to create machine learning algorithms that can accurately detect deepfakes, we must ensure the algorithm's design is to scale and able to process a high quantity of data for training and testing. Current computer vision and multi-modal algorithms based on deep learning architectures can meet these demands. The detection and reliability of these models, among other factors, is a direct result of the representativity of the training data. While it is easy to create deepfakes, the quantity of data required to train deepfake detection models is computationally prohibitive [31]. Therefore, it is essential to take advantage of existing datasets.

In [24], there are two distinguished generations of deepfake datasets. The first generation contains datasets such as DF-TIMIT [22], UADFV [62], and FaceForensics++ DF (FF++ DF) [19]. The second generation includes Google's Deepfake Detection Dataset [20], Celeb-DF [24], the Deepfake Detection Challenge Dataset Preview [63], among others.

One major challenge with these two generations of datasets is the limited number of swapped identities. Although the data quality improved from the first to the second generation, there is very little face-swapping within the datasets. This contributes to overfitting the model on those particular identities. Except for FF++ DF, fewer than 100 identities exist in these datasets. To minimize identities, the authors in Ref. [31] propose a third generation of datasets. This new generation of datasets contains a much larger set of frames and videos compared to previous generations. Moreover, these frames and videos are of higher quality, contain more identities within the content, and have consent from the individuals in the dataset. The following datasets fall within this generation: DeeperForensics-1.0 (DF-1.0) [64] and Deepfake challenge dataset (DFDC) [31].

Table 1 summarizes the generation of each dataset for comparison. It also indicates the following: the number of unique fake videos in each dataset and the total number of videos in each dataset; whether or not each dataset has secured image rights; the total number of subjects (identities) in each dataset; the number of methodologies used within each dataset; and the number of perturbations within each dataset. Each generation's respective data are as follows:

● **First Generation Datasets:** Most of the datasets are composed of 1000 videos or less, with less than 10^6^ frames in this generation. These datasets are composed of videos sourced from YouTube, however, there is no formal agreement or rights established over the identities depicted in the samples [65]. also indicated that most of the models trained on these datasets could not generalize to different deepfake generation methods.
● **Second Generation Datasets:** The second-generation datasets are composed of 1–10 thousand videos and 1 to 10 million frames. There is a significant improvement in the image quality compared to the first generation. Among these datasets, Celeb-DF is one order of magnitude larger than the previous generation. However, not all the material has been authorized those in the videos and frames. Therefore, this dataset raises ethical concerns about the image rights of use [66]. This dilemma led to the creation of Google's Deepfake Detection Dataset, created with 28 paid actors who authorized the use of their content. However, in the context of millions of videos, a composition of 28 identities would quickly generate overfitting problems on the models. Thus, the lack of variability in the dataset incurred generalization problems.
● **Third Generation Datasets:** The third generation contains datasets of the most recent releases in deepfake detection data. DF-1.0 and DFDC are composed of thousands of videos and millions of frames. Their human subjects were paid and consenting. Although belonging to the same generation, there are apparent quantitative and qualitative differences between the two datasets. In raw numbers, DF-1.0 is composed of 1000 unique videos, which were augmented by perturbations (overlays, geometric transformations, color transformations), increasing the dataset's size to a total of 60 thousand videos.

**Table 1:** Summary of deepfake detection datasets.

Dataset	Generation	Unique fake videos	Total Videos	Image Rights	Total Subjects	Number of Methods	Number of Perturbations
DF-TIMIT [22]	1	640	960	None	43	2	–
UADFV [62]	1	49	98	None	49	1	–
FF++ DF [19]	1	4000	5000	None	N/A	4	2
Google DFD [20]	2	3000	3000	Yes	28	5	–
Celeb-DF [24]	2	5639	6229	None	59	1	–
DF-1.0 [64]	3	1000	60 000	None	100	1	7
DFDC [31]	3	104 500	128 154	Yes	960	8	19

**Fig. 2:**
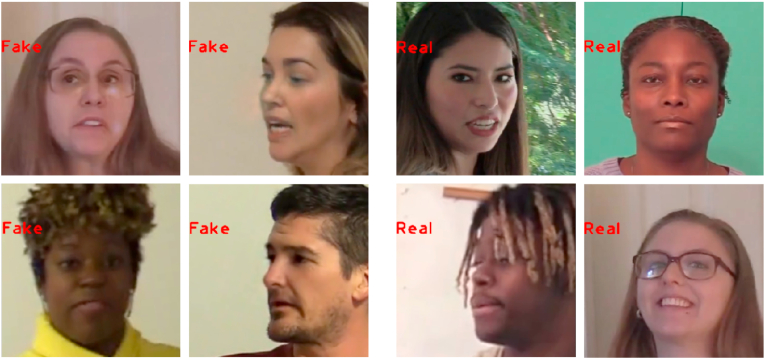
A sample of the faces extracted by our algorithm from the DFDC dataset and the true labels.

In DF-1.0, a single method is used to generate the face-swapping among the 100 actors. In contrast, DFDC has more than 100 thousand unique fake videos composed of the face-swapping of 960 different actors. To generate videos in the dataset, eight different creation methods were leveraged. Regarding the filming environment, DF-1.0 is at a disadvantage because all of its videos recorded in a studio, while DFDC has videos recorded indoors and outdoors. Example frames from DFDC are shown in Fig. 2.

## 3 Proposed architecture

**Fig. 3:**
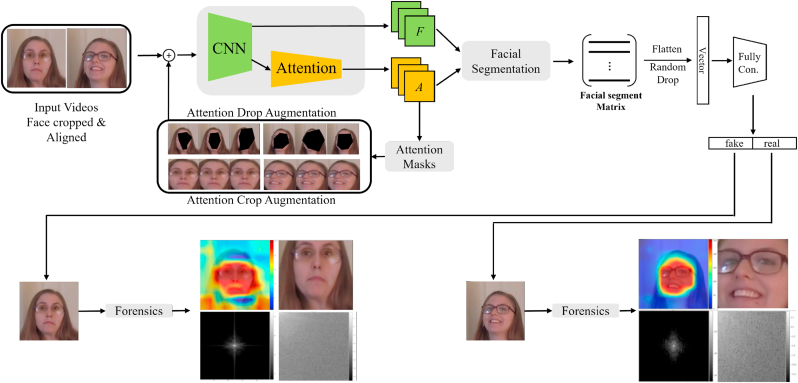
Our method incorporates an attention-based data augmentation.

Our method for hierarchical forensics of deepfake videos is a multi-step process, and is illustrated in Fig. 3. In this section, we formalize our proposed approach for deepfake detection and describe the individual components of our solution in detail. Our model processes an input video to generate the face inputs to the set of neural networks that we ensemble. We concatenate an ad-hoc explanation method that generates a heat-map highlighting the regions of the image that most contributed to the model's decision. We proceed with forensic analysis of this single highlighted region.

We address deepfake detection as a binary classification problem and propose a solution based on an explainable ensemble of CNN architectures. We further enhance the model by incorporating a weakly supervised deep attention data augmentation mechanism to process the feature maps of the classifier model. The attention-based data augmentation is not constrained to specific and prescribed labels, allowing for more flexibility in the learning process. This helps the model identify inconsistencies, irregularities, and invariances in the faces [67]. Our deepfake detection method allows the model to improve its perception of multiple regions of the face, preventing it from over-focusing on specific regions.

Furthermore, the attention mechanism enhances the model with a two-fold approach: *attention-based data augmentation* and *decision explanation*. Attention-based augmentation alters the data in a weakly supervised manner by simultaneously cropping (zoom) and dropping the attention regions of the classifier. The attention-based decision explanation highlights the critical regions for the user, generating heat maps that guide specialists to the decision attention points. Based on the discussion presented in Ref. [68], we further enhanced our classification method with the ensemble of 3 different networks. The heat map ranks regions of the image based on the importance of the decision. The model then crops the regions above a specified threshold from the image and generates deep forensic analysis to aid forensic specialists.

### 3.1 Data pre-processing

We train and evaluate our model on the DFDC dataset. The composition of the DFDC dataset consists of more than 128 thousand 10s short clips displaying one or more people in each video. Even though we generate a video-based prediction, our evaluation is on a frame-by-frame basis.

**Fig. 4:**
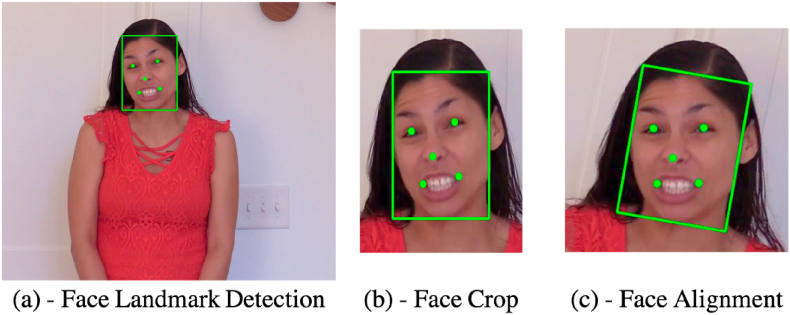
The frames extracted from the video are processed by the RetinaFace network, which (a) localizes the face and generates facial landmarks, (b) crops the region around the landmarks, and (c) performs an affine transformation to align the face based on the landmarks.

As the first step of our algorithm, we break the video into its constituent frames and sample 10% of these frames. Each extracted frame is pre-processed using RetinaFace [69]. RetinaFace is a robust single-stage face detector that performs pixel-wise face localization on various scales of faces. The basis of RetinaFace's network relies on extra-supervised and self-supervised multi-task learning. The algorithm performs three different face localizations. The first is face detection, the second is 2D face alignment, and the third is 3D face reconstruction based on a single shot framework. These three tasks are achieved with the goal of having the points regressed to lie on the image plane. Fig. 4 shows the pre-processing stages performed by RetinaFace to crop and align the face.

Once the faces were extracted and aligned, the output file is 320x320. Next, we used the mean and variance of the faces as normalization parameters in the network training, which ensured the input was standardized with little variance. Such normalization allows the training model to ignore compression and acquisition variations. Lastly, we enhanced our model's generalization by adding the following data augmentations: *horizontal flip, Gaussian Noise/ISO Noise, blur (motion blur and Gaussian blur), random hue-saturation modification, random brightness, contract modification, image sharpening and embossing, and lastly adding a sepia filter*. These augmentations are randomly applied in the training set.

### 3.2 Feature extraction and classification

Effective detection requires more than a single network. A survey among the best-rated solutions in the Deepfake Detection Challenge [31] shows that the ensemble of multiple networks leads to better accuracy.

We propose two different neural networks to extract features within an image for the detection of a deepfake image. Our overall decision combines the decisions of three networks: Xception [70], Xception + Augmentation, and Efficient-Net [71]+Augmentation. The previous section 3.3 discusses our augmentation of each network. These networks were selected based on how well they balance network size and accuracy. Furthermore, all three networks have an efficient operating and processing speed, and both have comprehensible network structures. Both structures use convolution layers that filter input to create a feature map that summarizes detected features in the input [72]. The convolution layers in these networks filter the image to identify the subject's characteristics (i.e. nose, hair, background). These filters are randomly initialized following a uniform distribution and optimized based on the dataset's data and labels. More specifically, the convolutional filters allow the network to uncover patterns and regions of the image (i.e. blurred lines, irregular facial symmetry, coarse features).

#### 3.2.1 Xception network

Researchers widely utilize the Xception network for deepfake detection due to its high accuracy and efficiency. The Xception network uses a variation of the Inception-Block [73] which is composed of depth-wise separable convolutions. Essentially, this model conducts a series of operations that independently look at cross-channel and spatial correlations [70]. Different from standard convolutions in which a single filter of depth *n* convolutes through the *n* channels of the input, a depth-wise convolution's operations are performed independently over each channel of the input and merged into a single volume by a 1-d filter of depth *n*, the point-wise convolution. This process reduces the required number of multiplications and parameters.

In many cases, this operation helps prevent overfitting without loss in the feature extraction process. The Xception architecture, however, inverts the order of operations. Instead of the point-wise convolution falling to the end of the process, it moves to the beginning, and locates itself before the depth-wise convolution. The inception module in Inception-v3 motivates this modification, such that the 1 × 1 convolution is accomplished before any *n* × *n* spatial convolutions. Within the Inception Network, there exists a non-linearity after the first operation; however, in Xception, there exists no intermediate non-linearity when considering depth-wise separable convolutions. In short, the Xception network linearly stack layers of depth-wise separable convolution and residual connections.

#### 3.2.2 EfficientNet-b3

The second network in our ensemble is the EfficientNet-b3 network. EfficientNets are used as an alternative to the standard layer stacking procedure that was first conceived in the ResNet architectures. He et al. identified that EfficientNet was more accurate and was less computationally expensive due to having fewer parameters. At the core of EfficientNet is its scaling methodology [74]. This architecture proposes a balance in the scale of multiple network parameters, such as depth, width, and resolution. Scaling CNNs in a mono-dimensional manner can result in rapidly deteriorating gains relative to its increased need for computational power. In the work of He et al., the skip connection allowed for the training of deeper layered nets, and ResNet has since been a very dominant architecture for computer vision [74]. By contrast, the same work shows that simply going deeper saturates these gains. Despite the advancements of ResNet, EfficientNet proposes a method to scale a CNN by incorporating width, depth, and resolution in the scaling of input, thus creating a multidimensional mechanism.

#### 3.2.3 Network ensemble

Ensembling is an older, yet highly effective technique to improve model generalization over the test set [68]. By employing a linear or nonlinear combination of the output of a few independent models over the same data, significant boosts of the ensembled model's overall accuracy occurred compared to the individual components. The benefits of an ensemble can be seen even when the training of the same architecture over the same dataset contains different initialization points. In our architecture, we ensemble the results of three different architectures: Xception, Xception + Attention, and EfficientNet-b3+Attention.

### 3.3 Weakly supervised attention network

A key task in improving the robustness of facial deepfake detection is extracting discriminative local features from multiple regions of the face. We utilize a weakly supervised attention-based data augmentation strategy, initially proposed in Ref. [75], for granular classification. This strategy enables a more objective learning process by allowing the model to re-evaluate the input image(Fig. 6(a)) under two new circumstances, one in which the most important attention region is removed from the image Fig. 6(b), and another in which the most important region is zoomed, Fig. 6(c). In order to obtain these regions, the data must undergo a weakly supervised attention augmentation process. Instead of requiring segmentation labels, convolutions generate attention maps that obtain the object's visual patterns, locations, and features. After obtaining the locations of facial segments, attention-guided data augmentation improves the model's feature extraction. This approach prevents the model from learning noise or random fluctuations in its respective training data (overfitting) on specific locations of a frame. Another benefit of the attention-guided data augmentation is that specific parts of the face are accurate, influencing the model to analyze objects more closely and refine its predictions.

**Fig. 5:**
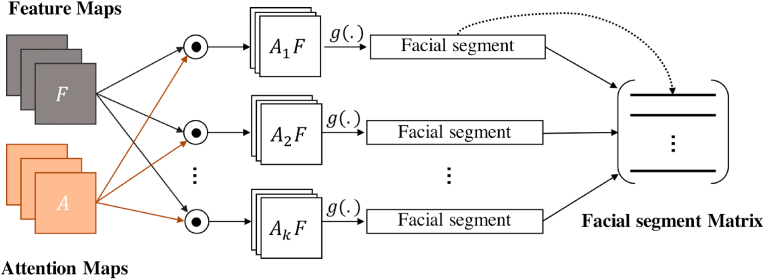
The feature maps and the attention maps are combined in a bilinear pooling operation, and stacked in the facial segment matrix.

**Fig. 6:**
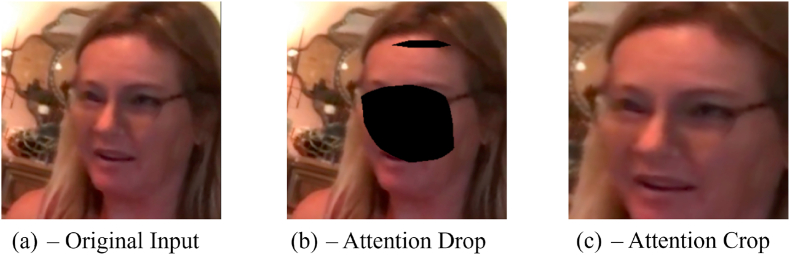
Based on the *k*^*th*^ attention map, a set of attention-based augmentation samples is generated.

#### 3.3.1 Spatial representation

We first predict the regions of objects during training and testing. Ultimately, the object's location annotations (e.g., bounding boxes or attention maps) are not available. Our method adopts weakly supervised learning to predict faces' location distribution based on the binary fake or non-fake prediction.

Given an input *X*, we extract the features, F(x), using the back-bone CNN model, in our case, Xception and EfficientNet-b3. The feature maps *F* ∈ *R*^*HxWxN*^ are representations extracted by the CNN model, in which *HxW* is the height and width of each feature map, and *N* is the number of maps extracted by the CNN architecture. Usually, these feature maps would be the single source of information passed to the fully-connected layers to make the model's decision. However, similar to granular classification, fake and real images share similar features, and very few details separate them from one another. This constraint can cause the model to be biased towards particular features and overlook others in classification. To improve the model's attention, we introduce an attention layer in the CNN networks. The output of the attention layer are the attention maps *A* ∈ *R*^*HxWxM*^, in which M is a hyper-parameter defining the number of attention maps. Formally, the definition of *A* is:

(1) $$A=f\left(F\right)=\overset{M}{\underset{k=1}{⋃}}A_{k}$$

In this equation, *f*(.) is a defined convolutional function composed of convolutional blocks (layers), batch-normalization (standardizes inputs to a layer), and non-linear activation function. Each *A*_*k*_ represents a point of attention within the input *X*. These points tend to be in regions around the forehead, mouth, and nose, as these regions often provide texture variations, asymmetry, and artifacts. To compose the embedding vector used for the decision, these attention maps are used to augment the training data and generate forensic indicators of manipulation. These indicators create the first step towards a robust and explainable deepfake detection methodology. Furthermore, using face locations discovered by attention maps enables end-to-end training in the binary deepfake detection task.

#### 3.3.2 Attention pooling

Attention maps are regions within the extracted features that act as points of interest. Based on Bilinear Pooling [76], these regions can be combined with feature maps to produce facial segments. An element-wise multiplication is executed between the feature maps and attention maps to obtain these facial segments. This multiplication is a set of *M* × *N* × *W* × *H* maps. With global average pooling function *g*(.), we average these maps to generate the facial segments. Fig. 5 demonstrates the facial segment matrix formation.

We expect that the attention map represents a similar facial segment for each category. With the use of center loss [77], we can weakly supervise the attention learning process. Center loss penalizes variance in the features of attention maps, such that the features should represent the same facial segment. With this penalty, we are pushing facial segments *f*_*k*_ to be closer to the global feature center *c*_*k*_ and pushing activation of the attention map *A*_*k*_ in the same facial segment. The expression of center loss is:

(2) $$L_{A}=\overset{M}{\underset{k=1}{\sum }}{\left\|f_{k}-c_{k}\right\|}_{2}^{2}$$

Thus, *c*_*k*_ is updated at every mini-batch using a the follow moving average formula:

$$c_{k}\leftarrow c_{k}+\beta \left(f_{k}-c_{k}\right)$$

### 3.4 Attention-based data augmentation

Even with large datasets, data augmentation can improve the model's robustness and generalization. However, randomized data augmentation can prove unhelpful. We provide two attention-based data augmentations: *attention cropping* and *attention dropping*.

#### 3.4.1 Attention cropping

Based on the attention map *A*_*k*_, we zoom into the attention region to acquire stronger and more detailed local features. We generate a mask *C*_*k*_ by comparing the elements of *A*_*k*_ with a threshold, marking 1 for those above the selected threshold. We then define the smallest bounding box that can fit the mask. This region is proportionally selected in the input image and enlarged to the input size. The generated image travels through the CNN pipeline as an extra input with the same label as *X*. Fig. 6 exemplifies.

#### 3.4.2 Attention dropping

Like attention cropping, attention drops rely on the *k*_*th*_ attention map to generate a mask. Unlike attention cropping, we use attention dropping to encourage the algorithm to look at other facial regions to generate its prediction. The algorithm is expected to drop the specific regions of the face and focus its attention during a specific training loop. This influences the CNN to extract different features for its decision. In contrast to attention cropping, we generate a mask by setting to 0 any element greater than the defined threshold. We apply the mask to the input *X*, and this new augmented input processes through the CNN pipeline. The elimination of the *k*_*th*_ segment of the face forces the network to evaluate other segments to generate its prediction, improving the robustness of the algorithm (as seen in Fig. 6).

### 3.5 Explainability

Undirected learning approaches have been proven to be the most effective in detecting deepfakes, provided enough data is available to train the model [31]. Although accurate, these approaches cannot be separated into individual intuitive components, making it challenging to interpret [18,78]. Considering our method is on par with human capabilities, it should be capable of sharing its findings and points of attention that have contributed to the decision. This visibility increases confidence in the model.

This algorithm's architecture aims to provide an interpretable decision that attributes a level of decision making importance to each input feature. *Feature attribution* is a known explainability method that explains a specific prediction by evaluating how much each feature input affects (positively or negatively) the model's decision. In this method, the features can range from tabular data to words. For the detection of deepfakes, we use pixel attribution. We based our explanation solely on the model's gradient, which indicates how changes in individual pixels affect the model's prediction. The method quantifies how much, and in which direction (positive or negative), small changes on specific pixels would impact the model's decision.

In order to generate explanations for deepfake detection, we use Grad-CAM [31], which can generate visual explanations for visual decisions. Using this method, the model's decision is propagated (through the gradients) back to a specified CNN layer, usually, the last convolutional layer. Our model considers the attention layer and the feature maps where the model does not focus its attention. Consequently, we evaluate the impact of these two layers on the model's decision by back-propagating the prediction into these layers and generating a coarse map that highlights the importance of the regions to the final model's decision.

**Fig. 7:**
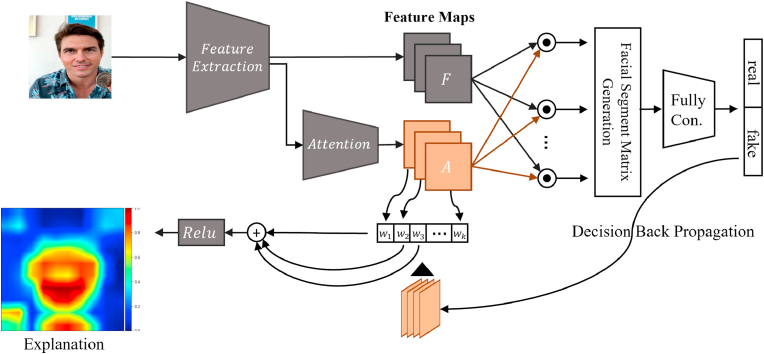
The explanation is based on the back propagation of the decision's loss to the attention maps. The gradient values for each attention map are used to weight the importance of each for the model's decision. The heat map is the weighted sum of the attention maps.

We have *N* feature maps and *K* attention maps in our model. Each of these maps contains information that impacts specific class outputs. These maps are high-dimensional abstract representations that include information relevant to all classes. Every pixel of all feature maps weight with the gradient concerning the output and the class of interest. The average of the weighted feature maps occurs, and each resulting pixel has a value ranging from −1 to 1. The values then pass through a ReLU activation function that will set every negative value to 0. The resulting heat-map is scaled and overlaid on the input image, generating visual interpretability, as shown in Fig. 7.

## 4 Experiments

### 4.1 Quantitative results

We evaluated our model using the DFDC test dataset, composed of 5000 short clips of 10s. Unlike the original training set, the test videos have distractors and noise applied. The objective of the perturbations is to simulate real-world conditions and added artifacts that may mislead automatic detectors, or even specialists. Our models make decisions on a frame-by-frame basis. To achieve the overall decision, we average the probabilities obtained for each frame.

As we can see in Table 2 and Table 3 the Xception + Attention model has a high accuracy and True Negative Rate when compared with the other two models. Xception and EfficientNet-B3+ Attention have lower accuracy when compared to the other models, but their addition in the form of ensemble raised the model's accuracy by a factor of almost 1%. This effect demonstrates how these models can specialize, having certainty in some decisions and uncertainty in others. When the averaging of decision probabilities occurs in a weighted manner, models with greater certainty will have more influence in the overall classification decision, resulting in a better performing model. In Fig. 9, we plot the receiver operating characteristic curves of the implemented models. We have also evaluated our models in an out-of-distribution setting, in which the model was presented a dataset it was not trained on. We find that the model maintains the same accuracy. The dataset proposed in Ref. [17] is composed of videos extracted direct from the internet, with no control over which algorithm has generated the video, or which compression algorithms these files went through. Such conditions make predictions much more difficult than in controlled datasets. Even under such harsh conditions, our algorithm was able to maintain an ROC above 0.8, indicating robustness against out of distribution samples (see Table 4).

**Table 2:** Quantitative evaluation of the detection models.

Model	Accuracy	True Positive	True Negative	False Positive	False Negative	AUC
Xception	83.88%	38.66%	45.04%	4.84%	11.2%	0.910 7
Xception + Attention	91.44%	45.39%	**45.86**%	4.02%	4.52%	0.972 4
EfficientNet-B3+ Attention	80.23%	**45.76**%	34.30%	15.58%	**4.14**%	0.916 0
Ensemble	**92.20**%	45.48%	46.52%	**3.36**%	4.42%	**0.975 2**

**Table 3:** Quantitative Evaluation of the Detection Models in out-of-distribution dataset CelebDF.

Model	Accuracy	True Positive	True Negative	False Positive	False Negative	AUC
Xception	89.37%	47.24%	42.18%	7.81%	2.81%	0.966 8
Xception + Attention	92.74%	47.01%	45.78%	4.21%	3.03%	0.978 4
EfficientNet-B3+ Attention	79.20%	43.08%	36.16%	13.83%	6.97%	0.880 8
Ensemble	93.64%	47.77%	45.86%	4.10%	2.24%	0.984 1

**Fig. 8:**
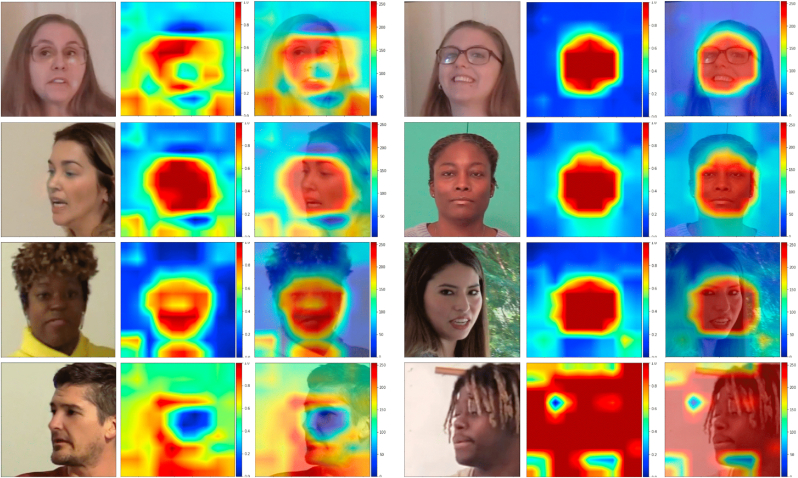
The left shows the explanations obtained by the GradCam algorithm when the fake prediction is propagated back to the attention layer. The right shows the explanation obtained from the real prediction, propagated back to the attention layer.

**Fig. 9:**
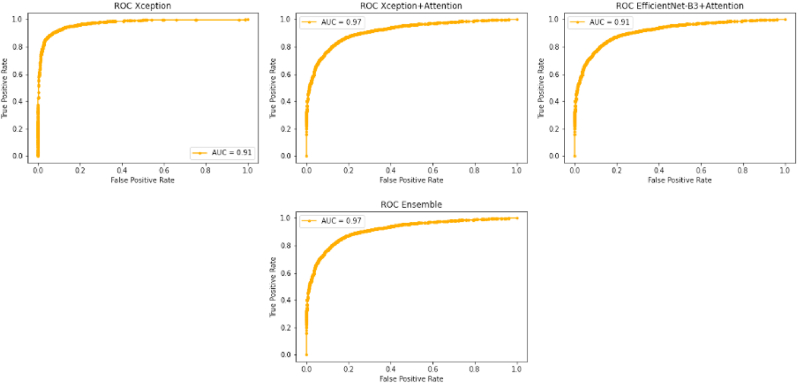
The receiver operating characteristic curves of the models.

**Table 4:** Quantitative Evaluation of the Detection Models in out of distribution dataset Wild Deep Fake.

Model	Accuracy	True Positive	True Negative	False Positive	False Negative	AUC
Xception	68.08%	42.55%	25.53%	27.12%	8.24%	0.780 0
Xception + Attention	71.33%	33.41%	37.91%	12.98%	1.56%	0.782 3
EfficientNet-B3+ Attention	54.55%	48.01%	6.54%	44.28%	1.15%	0.752 6
Ensemble	72.73%	35.90%	36.83%	15.82%	11.43%	0.807 9

### 4.2 Forensic deepfake detection and visualization

Given the black box nature of neural networks and the complexity of the calcuations, explaining these results can be nearly impossible, especially in a courtroom setting. To aid this dilemma, we visually conceptualize the machine's predictive analysis using a Grad-CAM approach to provide interpretable visual forensic explanations to the model's decision. Grad-CAM was ideal for our analysis, as it does not require a specific CNN architecture and is therefore highly versatile. Historically, multimedia forensics analyze an image's metadata (what is altered and left untouched), the Photo Response Non-Uniformity (fingerprint of the image), and the coefficient compression, to name a few [13]. If certain features or information were not present within the image, it would create suspicion that an image may be fake. Current neural network techniques (like those used in generating deepfake images) do not perform the same operations as traditional acquisition imaging sources.

By utilizing Grad-CAM, we exploit spatial information within convolutional layers to understand which parts of an image are necessary for the machine's classification decision. As seen in Fig. 8, the creation of localization maps, which highlight areas of importance within the image, provides the user insight into what features the algorithm had deemed significant with a heat map [18]. This method identifies critical areas that may contribute to the classification decision of a video or image by highlighting deepfake inconsistencies (blinking, warping, mouth placement) within the image space. Ultimately, this seeks to draw the user's attention to a region of the suspected deepfake.

### 4.3 Hierarchical forensic analysis

The results provided show that our model is very accurate under challenging circumstances, with failure in around 8% of cases. While the model's accuracy is comparable and superior in some cases to the state-of-the-art models, our model provides an extra level of confidence that is necessary for forensic applications. We analyzed the data points where our model provided a wrong prediction and concluded that 60% of these cases have a difference of less than 0.5 in probability to the true class. In contrast, most of the correct predictions predicted the true class with probabilities near 1. We integrate human intuition in our proposed solution by adding explanatory heat maps and by using the threshold heat map regions to crop an interest region of the face and generate a total frequency and statistical analysis. This method reduces the uncertainty of the predictions that do not output a probability near 1 because humans can review the heat maps and understand the model's decision. Furthermore, forensic analysis is not limited to the failed predictions, but can also be used to understand successful predictions.

**Fig. 10:**
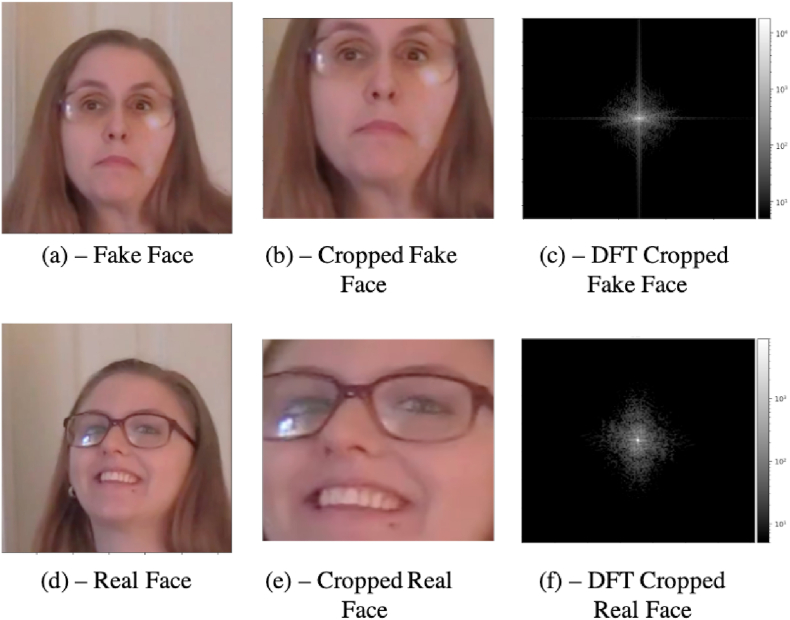
A comparison of the frequency analysis of a real and a fake sample shows the presence of abnormal frequencies.

This forensic analysis aims to provide analytical tools that lead to a conclusion about a specific video frame. We have initially evaluated the 2D Discrete Fourier Transform (DFT) of the region originated from the model's explanation. As seen in Fig. 10, the convolutional layers of the fake generation algorithm introduced abnormal frequencies in the face region.

We also consider an evaluation of the Discrete Cosine Transform (DCT) of the cropped images to evaluate if an image is a deepfake or not. In Ref. [79], it is suggested that the AC coefficients of the DCT follow a Laplace distribution, and the hyper-parameters *β*, estimated from a sample of real and fake images, can be used to distinguish between AC coefficients.

**Fig. 11:**
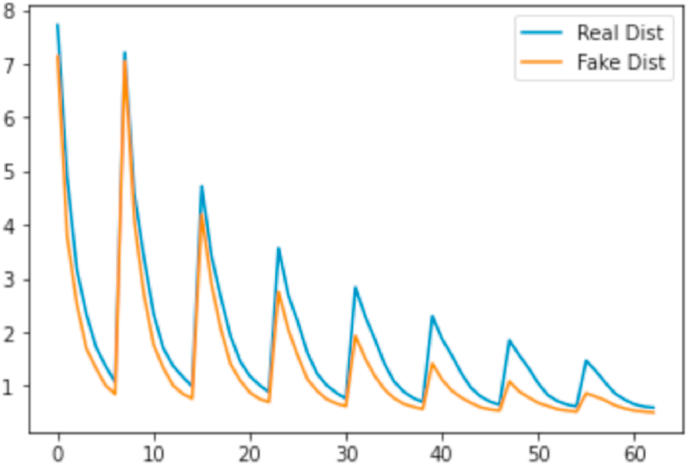
We plot the *β* statistics for fake and real samples. This is the average *β* value for the coefficient associated with each of the 63 frequencies.

We evaluated the cropped regions we extract based on the explanation maps concerning the Laplace distribution. Every image *X* was divided into non-overlapping blocks of size 8*x*8. We applied the DCT to every individual block and obtained 64 coefficients, the first being a DC component (frequency 0). The evidence reported in Ref. [79] describes the 63 AC components as:

$$P\left(x\right)=\frac{1}{2\beta }\exp\frac{-|x-\mu |}{\beta },$$

in which *μ* = 0 and $\beta =\frac{\sigma }{\sqrt{2}}$ (scaling factor). *σ* is the standard deviation of the AC coefficient distribution. We evaluated the distribution of the *β* coefficients for real and fake images. As seen in Fig. 11, the fake images have very similar statistics for *β* in the lower frequencies. This shows that the tendency does not follow higher frequency components that are used to identify traces of image manipulation.

## 5 Conclusion

This paper provided a thorough understanding of deepfake images and videos: how they are generated, how they can be detected, and how AI could be useful in their detection. We proposed an architecture that explores attention and feature maps to holistically investigate how well an AI can detect deepfake images using the same forensic techniques humans would use. We discovered that our proposed architecture is successful in detecting localized deepfake characteristics (i.e. anomalies in the face and jaw region). We also explored specific feature characteristics (eyes, nose, mouth) and learned that some features proved to be significant in detection. Our approach contributes to the field of deepfake detection by enhancing traditional forensic techniques with AI, and equipping the user with a deepfake detection methodology that is fast, comprehensible, and transparent.

## Financial disclosure

This material is based on research sponsored by the 10.13039/100000180Department of Homeland Security (DHS), United States Secret Service, National Computer Forensics Institute (NCFI) via contract number 70US0920D70090004.

## Declaration of competing interest

No Conflicts.URL https://search.informit.org/doi/10.3316/informit.807881125382837.

## Footnotes

1 Karen Hao, K. H.

2 FaceApp - AI Face Editor, https://apps.apple.com/us/app/faceapp-ai-face-editor/id1180884341.

3 Reface: Face Swap Videos, https://play.google.com/store/apps/details?id=video.reface.app.
